# Advances in thoracic ultrasound

**DOI:** 10.1183/13993003.00827-2025

**Published:** 2026-02-19

**Authors:** Dinesh N. Addala, Najib M. Rahman, Christian Dilling Kildegaard Andersen, Christian B. Laursen, Casper Falster

**Affiliations:** 1 Oxford University Hospitals NHS Foundation Trust, Department of Respiratory Medicine, Churchill Hospital, Oxford, UK; 2 Oxford Respiratory Trials Unit, Nuffield Department of Medicine, Oxford University, Headington, UK; 3 Oxford University Hospitals NHS Foundation Trust, Oxford NIHR Biomedical Research Centre, John Radcliffe Hospital, Headington, UK; 4 Department of Respiratory Medicine, Odense University Hospital, Odense, Denmark; 5 Odense Respiratory Research Unit (ODIN), Department of clinical research, University of Southern Denmark, Odense, Denmark; 6 Centre for Advanced Lung Cancer Diagnostics (CALU), Odense University Hospital, Odense, Denmark; 7 Department of Internal Medicine, Svendborg Hospital, Svendborg, Denmark

## Abstract

**Thoracic ultrasound (TUS) has revolutionised diagnostics and intervention in lung and pleural conditions. Recent data illustrate the utility of TUS in accelerating diagnostics in emergency and elective settings, while improving procedural safety.** https://bit.ly/4nY4mXc

## Introduction

Thoracic ultrasound (TUS) has rapidly transitioned from a bedside adjunct to a central component of modern respiratory and acute care medicine. Its ability to assess pleural and pulmonary pathology in real time, without radiation, has made it indispensable in managing pleural effusion, pulmonary congestion and acute dyspnoea. Recent evidence suggests TUS may not only confirm diagnoses but also direct treatment, improve procedural safety and enhance outcomes (table 1). In this article, we focus on selected areas where the clinical utility of TUS is expanding, specifically in heart failure, haemodialysis, pulmonary embolism and pleural disease. We do not address areas such as cardiac ultrasound and diaphragm scanning, which have been covered elsewhere in dedicated reviews.

**TABLE 1:** Summary of key clinical trials in thoracic ultrasound (TUS)-directed diagnosis and management

Trial	Clinical setting	Intervention	Comparator	Key outcomes
**CAVAL US-AHF [1]**	Acute heart failure (inpatient)	Lung and IVC ultrasound-guided diuretics	Standard care	↓ 90-day readmission/mortality (13.3% *versus* 36.7%)
**EPICC [2]**	Acute heart failure (inpatient)	Lung ultrasound-guided therapy (B-lines)	Standard care	No difference in outcomes Study underpowered
**Arvig *et al*. [3]**	Acute dyspnoea (ED)	Serial lung and cardiac ultrasound	Standard care	Faster symptom relief No difference in readmission or mortality
**CLUSTER-HF [5]**	Chronic heart failure (outpatient)	TUS-guided therapy at follow-up	Standard care	↓ Hospitalisations and urgent visits
**LUS-HF [6]**	Chronic heart failure (outpatient)	TUS-guided diuretic adjustment	Standard care	↓ Heart failure decompensation ↑ Functional status and QoL
**LUST [9]**	Haemodialysis	TUS-guided ultrafiltration and blood pressure management	Clinical assessment	↓ Pulmonary congestion No improvement in mortality/hospitalisation
**PRIME [15]**	Suspected PE (ED)	Multi-organ ultrasound (lung, heart, DVT)	Usual workup	↓ Need for CTPA by 45% Failure rate of 4%
**SIMPLE [25]**	Malignant pleural effusion	TUS-guided chest drain removal post-talc pleurodesis	Radiograph-guided	Shorter hospital stay (2 *versus* 3 days) Similar pleurodesis success

Trials are referred to by their abbreviated name, with further details in references. ED: emergency department; PE: pulmonary embolism; IVC: inferior vena cava; DVT: deep vein thrombosis; QoL: quality of life; CTPA: computed tomography pulmonary angiogram.

## Lung ultrasound

### Pulmonary congestion and heart failure

Cardiogenic pulmonary oedema is a leading cause of hospitalisation and mortality worldwide. Detecting pulmonary congestion early, and adjusting therapy accordingly, is central to management. TUS provides a dynamic, non-invasive means of quantifying congestion *via* B-lines, reflecting extravascular lung water. However, consensus on how to measure and act on B-lines is lacking, and this methodological heterogeneity may explain variable trial outcomes.

Several recent studies illustrate both the promise and challenges of TUS-guided therapy. The CAVAL US-AHF pilot randomised control trial (RCT) randomised 60 patients with acute decompensated heart failure to standard care *versus* TUS-guided therapy using B-lines and inferior vena cava (IVC) size. The TUS-guided group had significantly fewer residual congestion signs at discharge and markedly lower combined readmission or mortality at 90 days (13.3% *versus* 36.7%; p=0.038) [1]. While modest in scale, these results suggest that integrating TUS into diuretic titration can yield clinically meaningful outcomes.

By contrast, the EPICC trial, which aimed to recruit 152 patients but enrolled only 79, adjusted diuretics based on B-line counts at the treating clinician's discretion. It found no significant benefit in readmission or cardiovascular death [2]. In keeping with this, Arvig *et al.* [3] randomised 206 patients with acute dyspnoea to serial cardiopulmonary ultrasound *versus* standard care. In the subgroup with confirmed heart failure, TUS-guided care resulted in faster symptom relief but no reduction in readmission or mortality. The BLUSHED-AHF pilot also failed to show outcome benefit, though feasibility was demonstrated [4]. These trials highlight the importance of both study power and protocol fidelity; loosely standardised interventions risk diluting the effect of TUS.

In the outpatient setting, evidence is more consistent. The CLUSTER-HF trial enrolled 126 stable patients with chronic heart failure and found that routine TUS monitoring halved the rate of hospitalisations and urgent visits [5]. Similarly, the LUS-HF trial randomised 123 patients to TUS-guided diuretic adjustment *versus* standard care, reporting fewer decompensations and improved quality of life in the TUS arm [6]. These findings underscore the potential of TUS to detect subclinical congestion early and allow timely intervention, meeting a critical need in chronic heart failure management.

As the evidence of its utility continues to evolve, TUS is rapidly becoming an essential tool in managing cardiac pulmonary oedema across both hospital and outpatient settings. The subsequent increasing awareness is exemplified by the 2023 consensus statement from the European Association of Cardiovascular Imaging, which recognises TUS as a valuable modality for assessing pulmonary congestion in both acute and chronic heart failure [7]. The statement highlights TUS as an effective, non-invasive method for detecting subclinical congestion and guiding treatment strategies, positioning it as an essential component of contemporary heart failure management. Some variations in outcome in the inpatient setting may be attributable to the heterogeneity in protocols. Methods of assessment currently vary widely, from simplified anterior two-zone assessments to comprehensive 28-zone counts, and from IVC/B-line integration to the more complex VEXUS approach. The field as such requires a move to a consensus on reproducible, rapid protocols to assess fluid overload in future studies.

### Volume management in dialysis

In haemodialysis, achieving the correct “dry weight” is both essential and challenging. Overhydration contributes to hypertension and cardiac stress, while excessive ultrafiltration risks hypotension and organ hypoperfusion. TUS has emerged as a sensitive tool to assess fluid status by quantifying pulmonary congestion.

The LUST trial, a multicentre RCT of 363 patients, demonstrated that TUS-guided ultrafiltration reduced pulmonary congestion compared to clinical assessment alone. However, this did not translate into improved survival or hospitalisation outcomes [8]. Other trials provide complementary insights. Loutradis *et al*. [9] showed that TUS-guided dry weight adjustment significantly lowered ambulatory blood pressure and reduced intradialytic hypotension episodes. Corcoran *et al*. [10] found that TUS detected post-dialysis improvements in lung water more reliably than CT, with excellent interobserver agreement. More recently, Zachariah *et al*. [11] and Rendon-Ramírez *et al*. [12] provided further evidence that TUS improves accuracy of volume assessment, though again without hard end-point benefits.

The lack of mortality benefit does not diminish the practical value of TUS in dialysis. Reducing blood pressure variability and avoiding symptomatic hypotension represent important clinical outcomes. The likely explanation for the neutral primary end-points is that fluid status is only one of many contributors to outcomes in dialysis patients, alongside comorbidities, vascular access and infection risk.

### Community-acquired pneumonia

TUS has also gained recognition in the diagnosis of pneumonia. The 2025 American Thoracic Society guideline on community-acquired pneumonia endorsed TUS as an acceptable alternative to chest radiography for initial imaging in suspected cases, reflecting its high sensitivity for detecting consolidation and pleural involvement while avoiding radiation exposure [13]. Aggregated study populations suggested comparable or improved diagnostic accuracy *versus* chest radiography, particularly for peripheral lesions, although imprecision and methodological heterogeneity was noted in the majority of studies reviewed. While computed tomography (CT) remains the gold standard, in particular for complex cases, guideline inclusion highlights the growing role of TUS in point-of-care acute respiratory infection pathways and may be particularly useful in settings with lack of rapid access to radiography or CT imaging.

### Pulmonary embolism

TUS has been used to detect indirect signs of pulmonary embolism (PE), including wedge-shaped peripheral infarcts, right ventricular dysfunction, and lower limb deep vein thrombosis (DVT). A 2021 meta-analysis confirmed good diagnostic accuracy when multiple windows are combined [14].

The PRIME RCT, published in 2024, was the first to assess these in a clinical setting. Patients with suspected PE underwent lung, cardiac and DVT ultrasound. In cases with no ultrasound evidence of PE, referrals for CT pulmonary angiogram were reduced by 45% [15]. While this demonstrates the potential for ultrasound to streamline pathways and reduce unnecessary imaging, the 4% failure rate highlights the risks of relying on ultrasound as a sole rule-out test.

As such, TUS for PE may be utilised as a triage tool in selected settings, rather than as a replacement for definitive imaging. TUS may provide additional information to assist decision-making, but is unlikely to become the gold standard or replace CT imaging.

Emerging work on ultrasound-guided catheter-directed thrombolysis for sub-massive PE is in progress [16], and may represent an important future use of TUS. At present this technique is under assessment at specialist interventional centres and further data is required to inform on wider adoption.

### Pleural ultrasound

Pleural ultrasound is established as the safest method to guide aspiration and drain placement and is acknowledged as such in international guidelines [17]. The extent to which TUS can provide independent, clinically useful diagnostic information beyond procedural guidance has received much attention, with novel techniques emerging.

### Exudative and malignant pleural effusion

Data from the recent DUETS study suggested that TUS scoring systems may accurately differentiate transudative from exudative effusions, with a positive predictive value of 98.8% and negative predictive value of 100%, with a lower misclassification rate compared to Light's criteria [18]. This was, however, a single centre study and these findings require external validation before adoption into clinical practice. In our view, TUS remains a valuable complement rather than a replacement for biochemical testing, though it holds potential to reduce unnecessary procedures in clear-cut transudative cases if validated.

In malignant pleural effusion (MPE), TUS findings such as nodularity, thickening >1 cm, and echogenic fluid strongly correlate with malignant aetiology. Shiroshita *et al.* [19], in their 2020 meta-analysis, showed high specificity but only moderate sensitivity, confirming TUS as a useful rule-in but poor rule-out tool for diagnosing pleural malignancy. TUS therefore can prioritise patients for biopsy but does not replace the need for cytological or histological analysis, in particular with the increasing number of targeted oncological treatments available requiring molecular marker profiling.

Novel modalities are under evaluation. Shear wave elastography (SWE) measures tissue stiffness, with some studies reporting improved sensitivity over grayscale ultrasound for malignancy (84% *versus* 60%) [20]. SWE is, however, highly operator dependent, influenced by pleural thickness, probe pressure, and even respiratory motion, and currently lacks validated cut-off values [21].

Contrast-enhanced ultrasound (CEUS) highlights vascularised pleural lesions, but pleural-specific evidence remains minimal. One retrospective study suggested that the yield of ultrasound-guided pleural biopsy may increase with the use of CEUS, but the diagnostic yield without CEUS was lower than prior studies, and the cohort included a large number of patients with tuberculous pleural disease [22]. Our assessment is that both SWE and CEUS are promising tools in the assessment of pleural disease aetiology, but at present the data is insufficient to integrate them into routine clinical practice, in particular given the added equipment and training costs necessitated.

Ultrasound-guided cutting-needle biopsy offers a practical alternative for patients unsuitable for thoracoscopy. However, diagnostic yield (84%) remains consistently lower than for thoracoscopy (96%) [23], and molecular profiling success is also inferior [24]. With the growing role of pleural fluid supernatant in genotyping, the need for pleural biopsy in some (malignant) cases may be reduced. The authors would, however, consider thoracoscopy at present to be the gold standard technique for evaluation of undiagnosed pleural disease, and caution that invasive testing for pleural disease serves to diagnose conditions beyond malignancy alone.

### Emerging roles of thoracic ultrasound

TUS can be used to detect pleurodesis success in MPE. The SIMPLE trial randomised 313 patients to either usual care (using chest radiograph and drain output to guide chest drain removal following talc instillation) or TUS-directed drain removal. The median duration of admission was significantly shorter in the TUS arm (2 days *versus* 3 days in the usual care group; p<0.0001), with comparable 3-month pleurodesis success rates. The SIMPLE protocol involved a nine-point TUS assessment of lung sliding, and represents a time efficient, pragmatic advance that could be adopted widely with little extra training or equipment, while reducing requirements for repeated chest radiography [25].

TUS can also suggest non-expansile lung (NEL), with techniques such as M-mode and speckle-tracking imaging illustrating the potential to detect NEL non-invasively [26]. At present these represent areas of ongoing research that require wider assessment. The ability to reliably detect NEL prior to pleural intervention could lead to significant improvements in time to definitive intervention in MPE, through early triage to either indwelling pleural catheter or pleurodesis. We feel that this should remain a priority area in pleural ultrasound research.

Beyond pleural disease, TUS is increasingly being investigated in interstitial lung disease (ILD). Lung ultrasound can detect subpleural interstitial abnormalities with high sensitivity, and recent work suggests potential as a non-invasive screening tool for rheumatoid arthritis-associated ILD [27]. Multicentre studies are underway to assess reproducibility and clinical utility.

Artificial intelligence (AI) may further expand access to TUS by reducing inter-operator variability and supporting non-expert users. Pilot studies show that AI-assisted lung ultrasound can improve diagnostic confidence and enable non-specialists to obtain clinically useful images, even in resource-limited settings [28, 29]. Large-scale validation is still needed, but this remains a promising future direction.

## Conclusion

**FIGURE 1:**
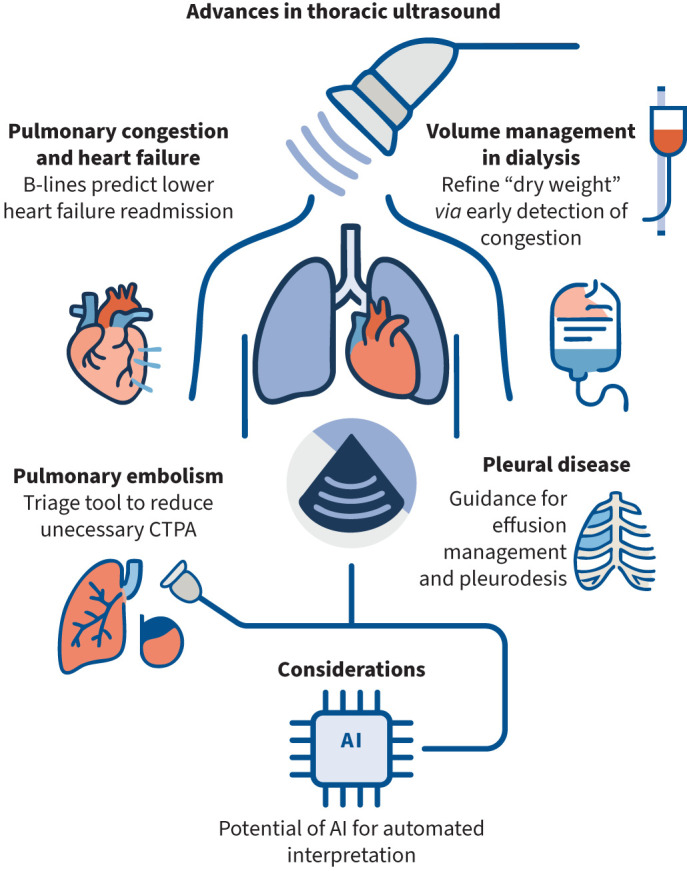
Summary of selected, key use cases of thoracic ultrasound and recent advances. CTPA: computed tomography pulmonary angiogram; AI: artificial intelligence.

TUS has become increasingly used in mainstream practice (figure 1), but its clinical impact varies by setting. In heart failure and fluid overload detection, outpatient use is supported by high-quality trial evidence and can be used to improve outcomes in specialist clinics. In PE, ultrasound may reduce unnecessary imaging but cannot be considered a definitive diagnostic strategy. In pleural disease, TUS is mandatory in undertaking safe procedures, useful for triage in malignancy, and effective in pleurodesis monitoring, but does reach limitations as a stand-alone diagnostic tool in the absence of pleural sampling.

The challenge for TUS lies in its wider adoption into regular clinical practice in a broader range of healthcare settings, including those with lower resources. In order to achieve this, standardisation, training and innovation are required. Consensus on scanning protocols, such as those to detect fluid overload, are essential to integration into clinical care. Evidence-based accreditation programmes, such as those from the European Respiratory Society [30], can ensure quality in practice and operator competence. Advancements in AI may assist with both elements, by reducing inter-operator variability, and facilitating access to TUS in areas with lower resources and access to training.

Despite these challenges, TUS has developed into an invaluable tool for delivering point of care diagnostics and rapid management, backed by increasingly high quality evidence to refine its optimal use cases.
